# Time-Dependent Behavior of Reinforced Polymer Concrete Columns under Eccentric Axial Loading

**DOI:** 10.3390/ma5112342

**Published:** 2012-11-16

**Authors:** Valentino Paolo Berardi, Geminiano Mancusi

**Affiliations:** Department of Civil Engineering, University of Salerno, Via Ponte Don Melillo 1, Fisciano (SA) 84084, Italy; E-Mail: g.mancusi@unisa.it

**Keywords:** reinforced polymer concretes, new construction, long-term behavior, numerical analysis, reliability

## Abstract

Polymer concretes (PCs) represent a promising alternative to traditional cementitious materials in the field of new construction. In fact, PCs exhibit high compressive strength and ultimate compressive strain values, as well as good chemical resistance. Within the context of these benefits, this paper presents a study on the time-dependent behavior of polymer concrete columns reinforced with different bar types using a mechanical model recently developed by the authors. Balanced internal reinforcements are considered (*i.e*., two bars at both the top and bottom of the cross-section). The investigation highlights relevant stress and strain variations over time and, consequently, the emergence of a significant decrease in concrete’s stiffness and strength over time. Therefore, the results indicate that deferred effects due to viscous flow may significantly affect the reliability of reinforced polymer concrete elements over time.

## 1. Introduction

Due to their lightness and high structural performance, polymer-based materials have been widely utilized in recent years to strengthen existing construction that are subject to static and seismic loads. Their general mechanical features [1] and constructive details [2] have been investigated in previous studies.

However, their use is still limited within the field of new construction due to the necessity of investigating critical aspects of their performance, such as those related to their reliability over time.

Within this context, polymer concretes (PCs) are a useful design option, because their strength is at least twice that of usual Portland cement-based concrete, their ultimate compressive strain is higher than that of cementitious concretes and they possess good resistance to chemical agents [3,4,5,6,7,8].

From a chemical point of view, PCs are composed of natural aggregates (e.g., silica sand or gravel) bound together with a thermoset resin. Consequently, they are particularly suitable for marine construction, tunnels, prestressed concrete members, and seismic applications because their ultimate compressive strain values allow for high ductility levels.

On the other hand, due to their limited tensile strength, PCs must be reinforced with either steel or FRP bars (e.g., GFRP or CFRP rods, which have already been utilized in ordinary reinforced concrete members in new structures [9,10]).

From an environmental point of view, the dioxins and furans generated by the manufacture of organic polymers are extremely toxic and bio-cumulative. Such substances have hazardous effects on biodiversity, contaminating every level of the food chain [11]. However, more sustainable manufacturing of these polymers has been achieved using combustion fly-ash and slag [12] or recycled PET waste [13,14,15,16,17,18,19,20,21].

At service conditions, it is well known that polymer-based materials can be characterized by significant deferred behavior [22,23,24,25,26], and relevant stress migrations have therefore occurred because these materials have been combined with more traditional materials affected by lower-viscosity flows [27,28,29,30,31,32].

Therefore, the differences between the rheological properties of the constituent materials in polymer-reinforced concretes could cause a relevant increase of the stress state in the reinforcement over time that is accompanied by an increase in displacements.

These aspects must be studied in more detail because they could affect the reliability of structural members at service conditions.

The few experimental and theoretical analyses available in the literature on this topic have highlighted the relevance of PC creep behavior without contributing any final conclusions, due to the approximations that affect these studies.

It should also be noted that the existing scientific investigations on this subject have typically considered only axial load cases, while eccentric axial load cases still require further analysis.

This paper presents a study on the time-dependent behavior of a polymer concrete column reinforced with different bar types under eccentric axial loading. The investigation is based on a mechanical model recently proposed by the authors in [32].

## 2. Mechanical Model

The mechanical model adopted in this study schematises the viscous behavior of a polymer concrete beam reinforced with either steel or FRP bars under combined bending and axial loading [32].

To briefly describe the theoretical background presented in [32], the main hypotheses and equations are given below.

The basic assumptions of the model are as follows:

- the generic plane cross-sections still lie within the plane after bending;
- perfect adhesion exists between the polymer concrete and rods;
- the external axial force, *N_ext_*, and the bending moment, *M_ext_*, are constant over time;
- there is no difference between the tensile and compressive stiffness/strength of a given material;
- the behavior of the polymer concrete is linear-viscoelastic;
- no cracks are present;
- the behavior of the reinforcement bars is linear-elastic;
- the internal reinforcing bars make no contribution to creep.

**Figure 1:**
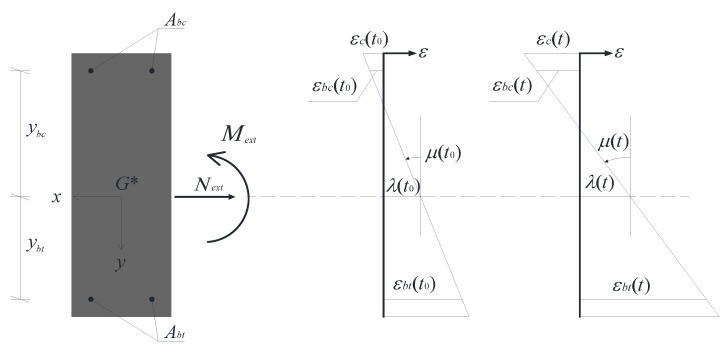
Instantaneous strain and curvature of the cross-section.

The following symbols are introduced in this paper:

- G* indicates the centroid of the transformed cross-section, assumed as the origin of the *x* and *y* axes (Figure 1);
- $\varepsilon_{c}$ and $\varepsilon_{b}$ denote the instantaneous values of the axial strain in the polymer concrete and the reinforcing bars, respectively, according to the following relationships (Figure 1):
  (1) $$\left\{ \begin{array}{l} \varepsilon_{c}\left(t,y\right)\;=\lambda \left(t\right)+\mu \left(t\right)y\;=\varepsilon_{ce}\left(t,y\right)\;+\varepsilon_{cv}\left(t,y\right)\\ \varepsilon_{b}\left(t,y_{b}\right)=\lambda \left(t\right)+\mu \left(t\right)y_{b}\;=\varepsilon_{be}\left(t,y_{b}\right) \end{array}\right.$$

where

- *λ*(*t*) and *µ*(*t*) are the axial strain at G* and the cross-sectional curvature, respectively;
- $y_{b}$ is the ordinate of the centroid of the generic bars;
- $\varepsilon_{ce}\left(t,y\right)$ and $\varepsilon_{be}\left(t,y_{b}\right)$ are the elastic polymer concrete and bar strains, respectively; and
- $\varepsilon_{cv}\left(t,y\right)$ is the viscous contribution to the polymer concrete strain.

We also define the normal stresses in the polymer concrete, *σ_c_*, in the top reinforcement bars, *σ_bc_*, and in the bottom bars, *σ_bt_*; the initial Young’s modulus of the polymer concrete,* E_c_*, and the reinforcement bars, *E_b_*; the polymer concrete area, *A_c_*, the overall top reinforcement rod area, *A_bc_*, and the overall bottom reinforcement rod area, *A_bt_*.

From Equation (1), the following expressions describing the stresses in both the polymer concrete and the bars can be defined as:

(2) $$\left\{ \begin{array}{l} \sigma_{c}\left(t,y\right)\;=E_{c}\cdot \varepsilon_{ce}\left(t,y\right)\;=E_{c}\left[\left(\lambda \left(t\right)+\mu \left(t\right)y\right)-\varepsilon_{cv}\left(t,y\right)\right]\\ \sigma_{bc}\left(t\right)=E_{b}\cdot \varepsilon_{be}\left(t,y_{bc}\right)=E_{b}\left[\left(\lambda \left(t\right)+\mu \left(t\right)y_{bc}\right)\right]\\ \sigma_{bt}\left(t\right)=E_{b}\cdot \varepsilon_{be}\left(t,y_{bt}\right)=E_{b}\left[\left(\lambda \left(t\right)+\mu \left(t\right)y_{bt}\right)\right] \end{array}\right.$$

The equilibrium equations of the cross-section can be expressed as:

(3) $$\left\{ \begin{array}{l} \int_{A_{c}}\sigma_{c}\left(t,y\right)dA_{c}+\int_{A_{bc}}\sigma_{bc}\left(t\right)dA_{bc}+\int_{A_{bt}}\sigma_{bt}\left(t\right)dA_{bt}=N_{ext}\text{ (translation)}\\ \int_{A_{c}}\sigma_{c}\left(t,y\right)ydA_{c}+\int_{A_{bc}}\sigma_{bc}\left(t\right)y_{bc}\;dA_{bc}+\int_{A_{bt}}\sigma_{bt}\left(t\right)y_{bt}\;dA_{bt}=M_{ext}\text{ (rotation about the }x\text{ axis)} \end{array}\right.$$

Using Equation (2), Equation (3) can be written in simple algebra as follows:

(4) $$\left\{ \begin{array}{l} E_{c}A^{*}\lambda \left(t\right)=E_{c}\int_{A_{c}}\varepsilon_{cv}\left(t,y\right)dA_{c}+N_{ext}\\ E_{c}I^{*}\mu \left(t\right)=E_{c}\int_{A_{c}}\varepsilon_{cv}\left(t,y\right)ydA_{c}+M_{ext} \end{array}\right.$$

where

- A^*^ = *A_c_* + *n_b_A_bc _*+ *n_b_A_bt_* is the area of the transformed section;
- I^*^ = *I_c_* + *n_b_I_bc _*+ *n_b_I_bt_* is the moment of inertia about the *x* axis of the transformed section;
- *n_b_* =* E_b_/E_c_*;
- *I_c_*, *I_bc_* and *I_bt_* are the moments of inertia about the *x* axis of the polymer concrete, the top reinforcement bars and the bottom reinforcement bars, respectively.

The viscous deformation $\varepsilon_{cv}\left(t,y\right)$ can be expressed as:

(5) $$\varepsilon_{cv}\left(t,y\right)=\int_{t_{0}}^{t}\frac{\sigma_{c}\left(\tau ,y\right)}{E_{c}}f_{c}\left(\tau ,t\right)d\tau$$

with

$$\begin{array}{lll} - & f_{c}\left(\tau ,t\right)=-E_{c}\frac{\partial \Phi_{c}\left(\tau ,t\right)}{\partial \tau } & \\ - & \Phi_{c}\left(\tau ,t\right)=\frac{1}{E_{c}}\left(1+\varphi_{c}\left(\tau ,t\right)\right) & \text{(creep function)}\\ - & \varphi_{c}\left(\tau ,t\right) & \text{(creep coefficient)} \end{array}$$

By accounting for the relationship in Equation (5), Equation (3) becomes:

(6) $$\left\{ \begin{array}{l} \lambda \left(t\right)=\left(\frac{A_{c}}{A^{*}}-1\right)\int_{t_{0}}^{t}\lambda \left(\tau \right)f_{c}\left(\tau ,t\right)d\tau +\frac{S_{c}}{A^{*}}\int_{t_{0}}^{t}\mu \left(\tau \right)f_{c}\left(\tau ,t\right)d\tau +\frac{N_{ext}}{E_{c}A^{*}}\left(1+\int_{t_{0}}^{t}f_{c}\left(\tau ,t\right)d\tau \right)\\ \mu \left(t\right)=\left(\frac{I_{c}}{I^{*}}-1\right)\int_{t_{0}}^{t}\mu \left(\tau \right)f_{c}\left(\tau ,t\right)d\tau +\frac{S_{c}}{I^{*}}\int_{t_{0}}^{t}\lambda \left(\tau \right)f_{c}\left(\tau ,t\right)d\tau +\frac{M_{ext}}{E_{c}I^{*}}\left(1+\int_{t_{0}}^{t}f_{c}\left(\tau ,t\right)d\tau \right) \end{array}\right.$$

Equation (6) represents a coupled system of two Volterra integral equations for the unknowns *λ*(*t*) and *µ*(*t*), which are solved using the Laplace transformation technique:

(7) $$\left\{ \begin{array}{l} L\left[\lambda \left(t\right)\right]=\left(\frac{A_{c}}{A^{*}}-1\right)\cdot L\left[\int_{t_{0}}^{t}\lambda \left(\tau \right)f_{c}\left(\tau ,t\right)d\tau \right]+\frac{S_{c}}{A^{*}}\cdot L\left[\int_{t_{0}}^{t}\mu \left(\tau \right)f_{c}\left(\tau ,t\right)d\tau \right]+\\ \;+\frac{N_{ext}}{E_{c}A^{*}}\frac{1}{s}+\frac{N_{ext}}{E_{c}A^{*}}\cdot L\left[\int_{t_{0}}^{t}f_{c}\left(\tau ,t\right)d\tau \right]\\ L\left[\mu \left(t\right)\right]=\left(\frac{I_{c}}{I^{*}}-1\right)\cdot L\left[\int_{t_{0}}^{t}\mu \left(\tau \right)\cdot f_{c}\left(\tau ,t\right)d\tau \right]+\frac{S_{c}}{I^{*}}\cdot L\left[\int_{t_{0}}^{t}\lambda \left(\tau \right)\cdot f_{c}\left(\tau ,t\right)d\tau \right]+\\ \;+\frac{M_{ext}}{E_{c}I^{*}}\frac{1}{s}+\frac{M_{ext}}{E_{c}I^{*}}\cdot L\left[\int_{t_{0}}^{t}f\left(\tau ,t\right)d\tau \right] \end{array}\right.$$

Assuming *t*_0_ = 0, *λ*(*t*) = 0 and *µ*(*t*) = 0 for *t* < *t*_0_, and* f_c_*(*τ,t*) = 0 for *t* < *τ*, the convolution theorem allows for the rewriting of the relationships in Equation (7) into the following form as an algebraic equation system:

(8) $$\left\{ \begin{array}{l} \lambda \left(s\right)=\left(\frac{A_{f}}{A^{*}}-1\right)\cdot \lambda \left(s\right)\cdot F\left(s\right)+\frac{S_{c}}{A^{*}}\cdot \mu \left(s\right)\cdot F\left(s\right)+\frac{N_{ext}}{E_{c}A^{*}}\cdot \frac{1}{s}+\frac{N_{ext}}{E_{c}A^{*}}\cdot \frac{F\left(s\right)}{s}\\ \mu \left(s\right)=\left(\frac{I_{f}}{I^{*}}-1\right)\cdot \mu \left(s\right)\cdot F\left(s\right)+\frac{S_{c}}{I^{*}}\cdot \lambda \left(s\right)\cdot F\left(s\right)+\frac{M_{ext}}{E_{c}I^{*}}\cdot \frac{1}{s}+\frac{M_{ext}}{E_{c}I^{*}}\cdot \frac{F\left(s\right)}{s} \end{array}\right.$$

where *F*(*s*),* λ*(*s*) and *µ*(*s*) represent the Laplace transforms of the functions *f_c_*(*τ,t*), *λ*(*t*) and *µ*(*t*), respectively.

The inverse Laplace transforms of *λ*(*s*) and *µ*(*s*) provide a solution to the viscous problem in the time domain.

## 3. Numerical Analysis

A numerical investigation of a polymer concrete column strengthened with several bar types is developed in this study. Only balanced internal reinforcements are accounted for and are composed of two bars at both the top and bottom of the cross-section.

Different rods are considered, the geometrical and mechanical properties of which, as certified by the manufacturer, are given in Table 1. The symbols *Φ_b_*, *E_b_* and *f_b_* denote the diameter, longitudinal Young’s modulus and strength of the bar, respectively.

Table 2 lists the ingredients of the polymer concrete under study, its longitudinal Young’s modulus, *E_c_*, and its strength,* f_c_*. These properties correspond to those obtained experimentally for the GFA 45 polymer concrete in [12].

**Table 1:** Certified geometrical and mechanical properties of the reinforcement bars.

*Bar ID*	*Material*	*Φ_b_* (mm)	*E_b_* (GPa)	*f_b_* (MPa)
SI	Steel	8	210.00	450.00
SII	Steel	10	210.00	450.00
SIII	Steel	12	210.00	450.00
SIV	Steel	16	210.00	450.00
CI	CFRP	8	115.00	2000.00
CII	CFRP	10	115.00	2000.00
CIII	CFRP	12	115.00	2000.00
CIV	CFRP	16	115.00	2000.00
GI	GFRP	8	40.00	1000.00
GII	GFRP	10	40.00	1000.00
GIII	GFRP	12	40.00	1000.00
GIV	GFRP	16	40.00	1000.00

**Table 2:** Composition and mechanical properties of the polymer concrete (type GFA 45 in [12]).

Binder Type	Mix Fraction (% of aggregate to polymer binder)	*E_c_* (GPa)	*f_c_* (MPa)
Alkali sodium silicate-activated coal, combustion fly ash and slag	23% Fine natural dune sand 29% 9 mm greywacke 18% 14 mm greywacke	10.00	41.50

**Figure 2:**
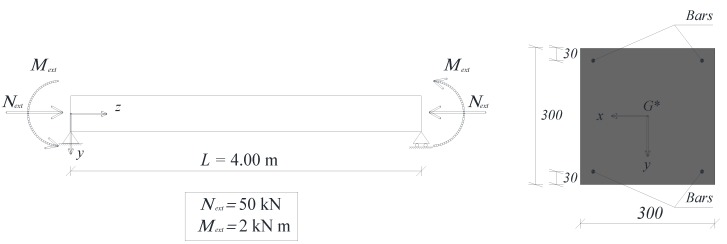
Reinforced polymer concrete beam (cross-sectional dimensions in mm).

**Figure 3:**
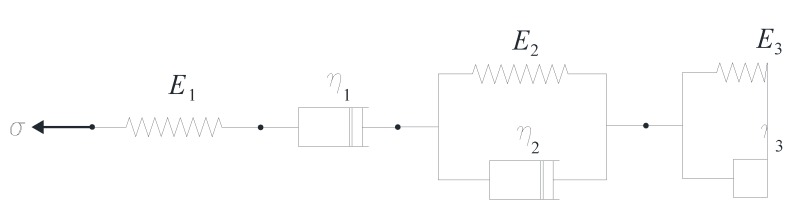
Bruger–Kelvin model.

The geometry, boundary conditions and loads used in the analysis are shown in Figure 2. The creep behavior of the polymer concrete is simulated with the Bruger–Kelvin viscoelastic model (Figure 3).

The viscous properties of the polymer concrete are evaluated from the experiments reported in [12]. To this end, the experimental data are fitted using the ordinary least-squares method, limiting the analysis to the secondary creep range due to its relevance when dealing with the service life of civil structures. The rheological parameters considered in the present study are listed in Table 3.

**Table 3:** Rheological properties of the polymer concrete (type GFA 45 in [12]).

*E_1_* (GPa)	*E_2_* (GPa)	*E_3_* (GPa)	*η_1_* (GPa d)	*η_2_* (GPa d)	*η_3_* (GPa d)
7.38	0	0	5.47 × 10^2^	0	0

The analysis is carried out with reference to the beam’s end cross-section (*i.e.*, at *z* = 4.00 m), accounting for the reinforcement types indicated in Table 1.

**Figure 4:**
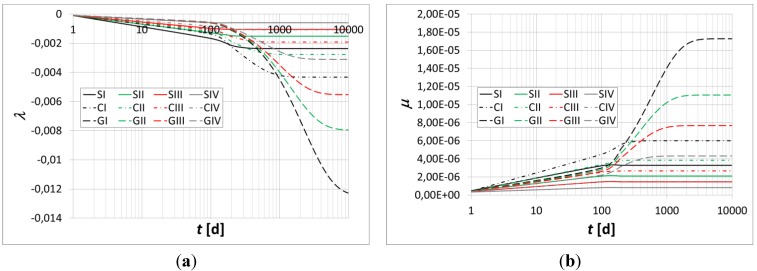
(**a**) Axial strain* vs.* time; (**b**) Curvature* vs.* time.

**Figure 5:**
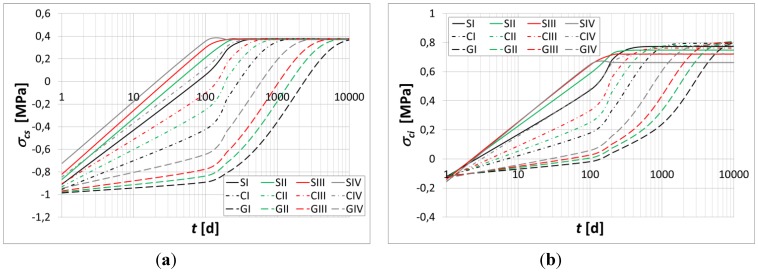
(**a**) Instantaneous stresses at the top fiber of the beam; (**b**) Instantaneous stresses at the bottom fiber of the beam.

**Figure 6:**
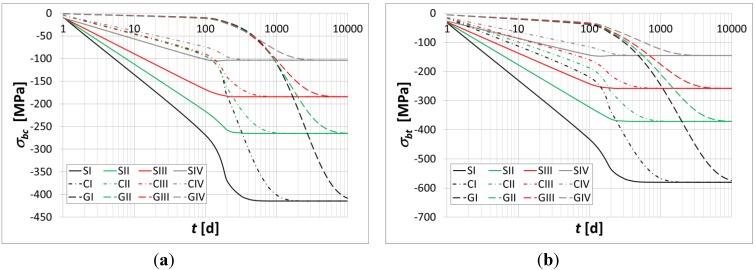
(**a**) Instantaneous stresses in the top reinforcement bars; (**b**) Instantaneous stresses in the bottom reinforcement bars.

The flexural curvature, axial strain, normal stresses at the top fiber, *σ_cs_*, and bottom fiber, *σ_ci_*, of the beam, and normal stresses in the top and bottom reinforcement bars are evaluated over time in Figure 4, Figure 5 and Figure 6.

The main results are summarized in Table 4 and Table 5. In particular, the percentage variations in the axial strain, curvature and stresses in the constituent materials are evaluated at the time instant *t* = 10000 h.

It should be noted that the initial stresses in the polymer concrete are less than 40% of the corresponding failure strength. This requirement is pivotal to satisfying a basic assumption of the linear viscoelasticity theory.

Moreover, the results obtained show a substantial increase in the axial strain, curvature, and relevant stress migration from the concrete to the bars due to the relevance of the polymer concrete’s creep flow.

**Table 4:** Instantaneous stresses in the polymer concrete.

*Bar ID*	*σ_cs_*(0) (MPa)	*σ_cs_*(10000 d) (MPa)	∆*σ_cs_*/*σ_cs_*(0) (%)	*σ_cs_*(0) (MPa)	*σ_cs_*(10000 d) (MPa)	∆*σ_cs_*/⎕*σ_cs_*(0) (%)
SI	−0.93	0.38	−140.57	−0.14	0.77	−643.20
SII	−0.90	0.38	−141.90	−0.16	0.75	−579.62
SIII	−0.87	0.38	−143.39	−0.17	0.72	−524.66
SIV	−0.79	0.38	−146.65	−0.19	0.66	−441.15
CI	−0.96	0.38	−139.43	−0.13	0.80	−714.37
CII	−0.94	0.38	−140.21	−0.14	0.78	−663.48
CIII	−0.92	0.38	−141.13	−0.15	0.76	−614.35
CIV	−0.87	0.38	−143.27	−0.17	0.72	−528.72
GI	−0.99	0.37	−137.13	−0.12	0.80	−779.79
GII	−0.98	0.38	−138.64	−0.12	0.1	−764.35
GIII	−0.97	0.38	−139.10	−0.13	0.80	−737.21
GIV	−0.95	0.38	−139.98	−0.14	0.78	−677.76

**Table 5:** Instantaneous stresses in the reinforcement bars.

*Bar ID*	*σ_cs_*(0) (MPa)	*σ_cs_*(10000 d) (MPa)	∆*σ_cs_*/*σ_cs_*(0) (%)	*σ_cs_*(0) (MPa)	*σ_cs_*(10000 d) (MPa)	∆*σ_cs_*/*σ_cs_*(0) (%)
SI	−6.30	−414.47	6473.95	−24.34	−580.25	2284.10
SII	−6.54	−265.26	3941.06	−23.56	−371.36	1476.37
SIII	−6.82	−184.21	2602.65	−22.69	−257.89	1036.53
SIV	−7.23	−103.62	1333.60	−20.82	−145.06	596.60
CI	−3.21	−414.47	12401.80	−13.70	−580.25	4134.80
CII	−3.41	−265.26	7676.02	−13.44	−371.36	2662.20
CIII	−3.51	−184.21	5141.24	−13.15	−257.89	1861.59
CIV	−3.72	−103.62	2684.02	−12.47	−145.06	1063.74
GI	−1.11	−408.40	36703.60	−4.88	−574.19	11674.90
GII	−1.12	−264.93	23483.60	−4.84	−371.03	7561.91
GIII	−1.14	−184.20	16066.90	−4.80	−257.88	5270.21
GIV	−1.18	−103.62	8705.11	−4.70	−145.06	2298.44

## 4. Conclusions

This paper presents an investigation on the long-term behavior of a polymer concrete column reinforced with several bar types. The analysis is carried out using the mechanical model proposed previously [32].

The numerical simulations highlight relevant stress migration from the polymer concrete toward the reinforcement bars in the analyzed beam.

A very relevant increase in the stresses within the top rods and those within the bottom rods is observed at the time instant *t* = 10000 h.

Moreover, a significant decrease in the top and bottom peak stresses of the polymer concrete and a substantial increase in the displacements of the beams’ long-term configurations are noted due to the high variation in their axial strain and flexural curvature.

The results also allow the following remarks to be made:

- the stress variations over time within the reinforcement bars may lead to the failure of a structural member because the stresses in the rods approach the available strength (see the analysis concerning a beam reinforced with 8-mm steel bars), thus compromising the safety of the entire structure;
- the long-term displacements of a structural member may produce, in hyperstatic frames, noticeable stress redistributions due to the continuity conditions, thus affecting the stress state in the entire structure;
- better deferred behavior of a reinforced polymer concrete column can be expected as the Young’s modulus and overall area of the internal rebars increase.

Finally, the current study highlights that the stiffness and strength of beams made from reinforced polymer concrete can change considerably over time due to viscous phenomena. This situation requires an accurate evaluation over time of the stresses, deflections and cracks at the serviceability limit state, which is mandatory to assess the beams’ reliability over time.
